# Is There an Optimal Spacer Cation for Two-Dimensional Lead Iodide Perovskites?

**DOI:** 10.1021/acsmaterialsau.4c00101

**Published:** 2024-11-26

**Authors:** Jiazhen Gu, Yongping Fu

**Affiliations:** † Beijing National Laboratory for Molecular Science, State Key Laboratory of Rare Earth Materials Chemistry and Applications, College of Chemistry and Molecular Engineering, Peking University, Beijing 100871, China

**Keywords:** Two-dimensional perovskites, Structural distortions, Exciton−phonon coupling, Photoluminescence quantum yield, Exciton diffusion

## Abstract

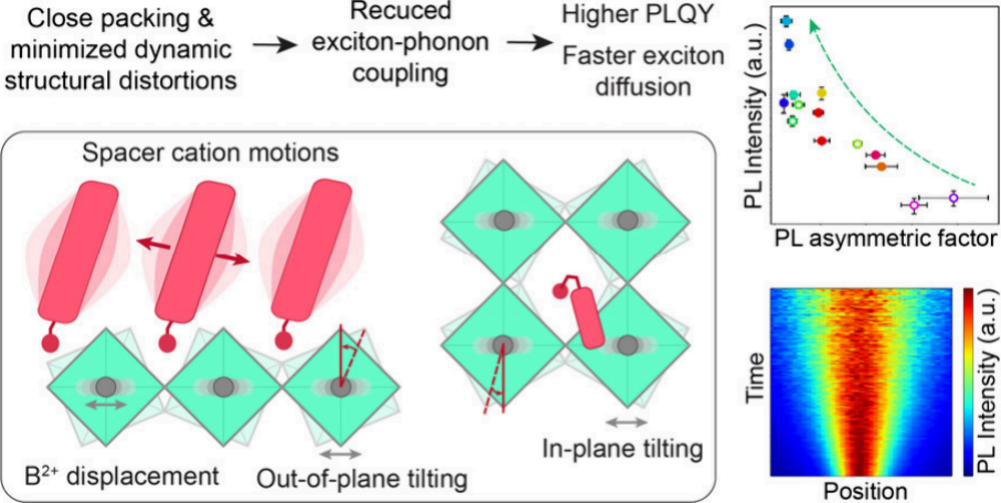

Two-dimensional lead iodide perovskites have attracted significant attention for their potential applications in optoelectronic and photonic devices due to their tunable excitonic properties. The choice of organic spacer cations significantly influences the light emission and exciton transport properties of these materials, which are vital for their device performance. In this Perspective, we discuss the impact of spacer cations on lattice dynamics and exciton–phonon coupling, focusing on three representative 2D lead iodide perovskites that exhibit distinct types of structural distortions. Minimizing structural distortions, such as dynamic out-of-plane octahedral tilting and lone pair distortion, appears to be essential for achieving narrow photoluminescence (PL) emission peaks, high PL quantum yields, and rapid exciton diffusion by suppressing exciton–phonon coupling, as demonstrated in 2D perovskites based on phenylethylammonium cation or its derivatives. We propose that designing spacer cations with enhanced intermolecular interactions and denser packing, combined with the close packing of inorganic ions to minimize the motions of both organic and inorganic lattices, would be the ideal scenario for yielding the most favorable optoelectronic properties in these materials.

## Introduction

Two-dimensional (2D) organic–inorganic hybrid metal halide perovskites have emerged as promising semiconductors for applications in light emitting diodes (LEDs), lasers, and other optoelectronic and spintronic devices.^1−4^ These materials consist of periodic stacks of organic and inorganic 2D layers, forming a quantum-well structure. The organic layers typically exhibit a large energy gap between the highest occupied molecule orbital (HOMO) and the lowest unoccupied molecular orbital (LUMO), leading to type-I band alignment that confines charge carriers within the inorganic layers.^5^ Due to strong quantum and dielectric confinement effects, excitons with binding energies reaching hundreds of meV dominate the optical and electronic properties of these materials. Compared to other 2D semiconductors, the versatility of 2D perovskites lies in the wide tunability of their excitonic properties through the selection of organic molecules, as well as the ease with which exotic properties can be incorporated, making them suitable for exploring excitonic physics and devices.^1,4,6,7^ For example, the incorporation of chiral organic molecules can produce chiral and spin-polarized excitons, offering potential for chiroptoelectronics and spintronics.^1,4^ Recent advancements have shown that LEDs based on 2D perovskites can achieve external quantum efficiencies of up to 20%,^8^ on par with state-of-the-art 3D perovskites and nanocrystals of conventional inorganic semiconductors. Furthermore, these materials exhibit strong light-matter interactions, with Rabi splitting energies reaching several hundred meV in optical microcavities, making them promising candidates for room-temperature exciton-polariton condensates and quantum devices.^9,10^

Fulfilling the potential of 2D perovskites, however, demands a comprehensive understanding of the structure–property relationships that can guide design and optimization of these materials. The simplest crystal structure of the 2D perovskites can be conceptualized by slicing a single layer of corner-sharing octahedra from a 3D perovskite (ABX_3_, where A^+^ is a small cation, B^2+^ is a divalent metal cation, and X^–^ is a halide anion) and replacing the small A-cation with a large organic ammonium (LA^+^) spacer cation, resulting in the general formula (LA)_2_BX_4_. This Perspective focuses on the cases of single-layer 2D lead iodide perovskites. While the spacer cations do not directly contribute to the band edge states, the choice of organic cations influences structural distortions of the inorganic layers, thereby altering the band structures and exciton behaviors. Moreover, the optoelectronic properties are significantly influenced by exciton–phonon coupling, which imparts a pronounced polaronic character to excitons in 2D perovskites,^11−14^ thereby altering the excited-state energy landscape. The exciton–phonon coupling is further modulated by the choice of spacer cations due to strong interaction between the vibrational modes of the organic and inorganic layers.^15−18^ The successful incorporation of a spacer cation into 2D perovskite structures appears to be only constrained by its cross-sectional area,^19,20^ leading to the use of a variety of aliphatic and aromatic ammonium spacer cations. To date, several hundreds of crystal structures have been identified in the crystallographic database, showing significant variations in photoluminescence (PL) and exciton transport properties.^21−34^ These variations raise an interesting question: *Is there an optimal spacer cation that can yield the most favorable optoelectronic properties?* Addressing this question requires a deep understanding of how the spacer cations influence structural distortion and exciton–phonon coupling, particularly concerning PL quantum yield and exciton diffusivity, which are key parameters in determining the performance of optoelectronic devices.

## Structural Distortions and Light-Emitting Properties

The structural distortions in 2D perovskites are influenced by several factors related to the spacer cations, including size, conformation, steric effect, packing distance, dynamic disorder, and hydrogen bonding with the inorganic layer. Since 2D perovskites share the same corner-sharing metal halide octahedra as their 3D counterparts, similar type of structural distortions can be expected.^2^ In 3D perovskites, the structural distortions are governed by the relative radii of the A–, B–, and X–site ions, which can be empirically predicted using the Goldschmidt tolerance factor, 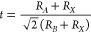
where *R*_*A*_, *R*_*B*_, and *R*_*X*_ are the ionic radii of the A–, B–, and X–site ions, respectively. When *t* = 1, the perovskite structure is ideally cubic, with closely packed ionic spheres. However, deviations from *t* = 1 lead to structural distortions due to instability at either A–site or B–site.^35,36^ Specifically, when *t* < 1, the A-site cations are undersized for the cavity formed by eight [BX_6_] octahedra, typically resulting in octahedral tilting. This tilting reduces the void space and strengthens ionic bonding between the A–cations and metal halide framework, as observed in perovskites such as MAPbI_3_ (MA^+^ = methylammonium) and CsPbI_3_. On the other hand, when *t* > 1, the A–site cation is oversized, causing lattice expansion and stereochemical expression of the n*s*^*2*^ lone pair on the B–site cation (i.e., Pb^2+^, Sn^2+^, or Ge^2+^). Similar to lead chalcogenides, the Pb–I bond exhibit partial covalency.^37^ The stereochemical expression of n*s*^*2*^ lone pair is a second-order Jahn–Teller distortion, driven by an increase in covalency from the viewpoint of molecular orbital interactions.^38^ A key manifestation of this lone pair expression is the off-center displacement of the B–site cation, which occurs when it results in an energy gain through the mixing of ground and excited electronic states in the distorted octahedral configuration.^39^ This type of distortion (also referred to as lone pair distortion) is typical in perovskites such as MHyPbBr_3_ (MHy^+^ = methylhydrazinium) and MAGeI_3_,^40^ and serves to maximize covalent bonding between the lead and halide atoms.

It should be noted that even perovskites with *t* deviating from 1 may exhibit nominally cubic structures at high temperatures, which are the average result of dynamic and/or static distortions.^41,42^ We are not aware of a 3D halide perovskite with a single A-cation achieves a perfectly cubic close-packed structure. Here, “a perfectly cubic close-packed structure” refers to a structure that retains cubic symmetry even at low temperatures. In this context, close packing implies no free space around both the A–site and B–site cations, which would prevent phase transitions upon cooling. While many 3D halide perovskites belong to cubic space groups at higher temperatures, these ions are not close-packed, as evidenced by their phase transitions into lower-symmetry space groups at lower temperatures.^43^ Alloying different A-cations to adjust the apparent tolerance factor could potentially stabilize the cubic structures at low temperatures. Indeed, the best-performing perovskite solar cells are based on compositions with alloyed A-cations, which help optimize the structural and electronic properties for enhanced performance.^44^

**Figure 1:**
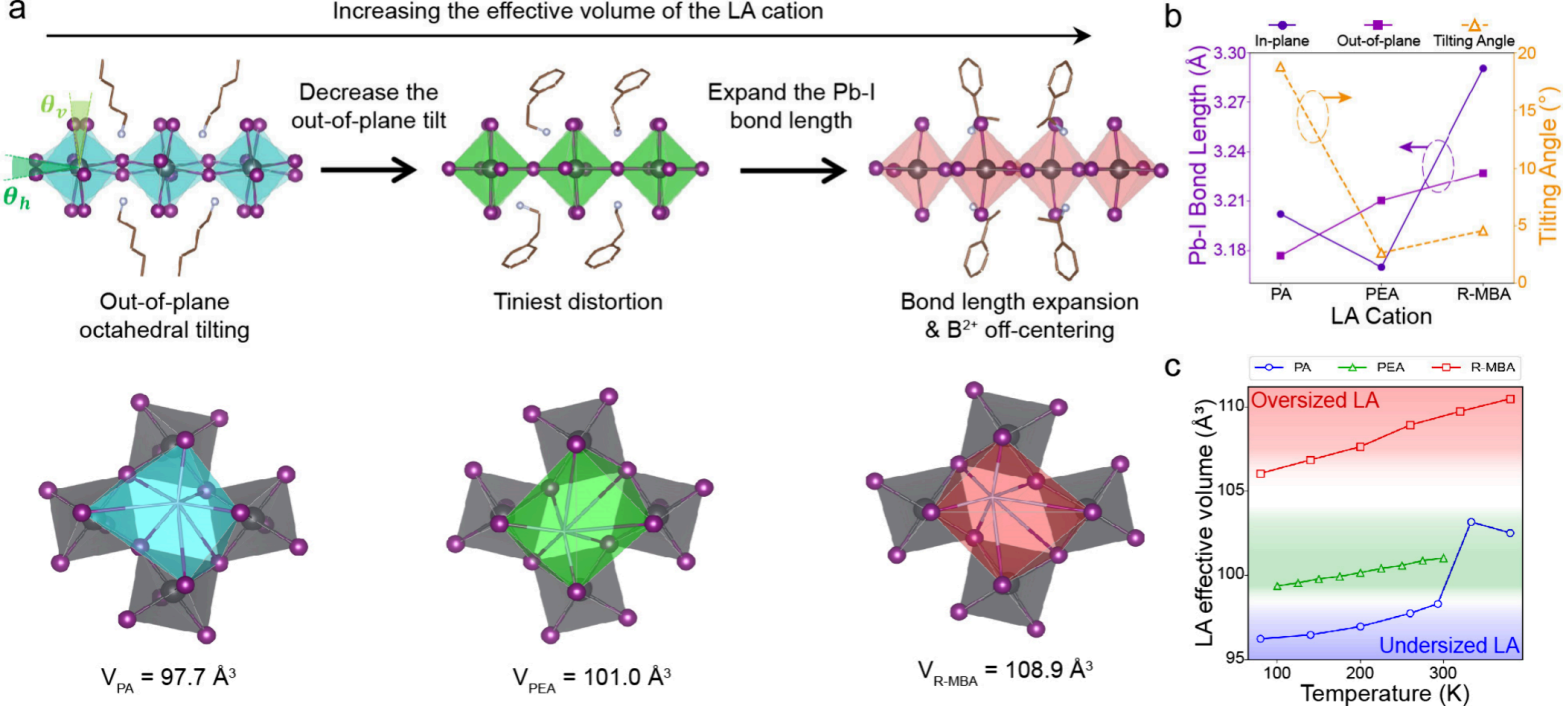
Structural distortions in three representative 2D lead iodide perovskites. (a) The evolution of structural distortions as the steric hindrance of the spacer cation (LA^+^) increases in the three archetypal structures: (PA)_2_PbI_4_, (PEA)_2_PbI_4_, and (R-MBA)_2_PbI_4_. Out-of-plane octahedral tilting angles (*θ*_*n*_*,θ*_*v*_) are defined as the I–Pb–Pb–I dihedral angles between adjacent octahedra. The effective volumes of the LA^+^ cations are defined as the volumes of the [NI_8_] polyhedra, depicted in different colors for the three structures. (b) Average Pb–I bond lengths and out-of-plane tilting angles in (PA)_2_PbI_4_, (PEA)_2_PbI_4_, and (R-MBA)_2_PbI_4_. Purple dots and squares indicate the Pb–I bond lengths along the in-plane and out-of-plane direction, respectively. Orange triangles indicate the average octahedral tilting angles, calculated as (*θ*_*n*_*,+θ*_*v*_)/2. (c) Effective volumes of PA^+^, PEA^+^, and R-MBA^+^ at different temperatures. Reproduced with permission from ref (22). Copyright 2023 Wiley-VCH GmbH.

Analysis of the extensive crystal structures of (LA)_2_PbI_4_ in the crystallographic database reveals that the packing of the spacer cations dictates the structural distortions in 2D perovskites.^22^ Specifically, loose packing associated with an “undersized” spacer cation results in out-of-plane octahedral tilting, whereas compact packing of an “oversized” spacer cation stretches the Pb–I bond length, leading to Pb^2+^ off-center displacement driven by the stereochemical expression of 6*s*^2^ lone pair. Figure 1a shows three representative crystal structures: (PA)_2_PbI_4_ (PA^+^ = pentylammonium), (PEA)_2_PbI_4_ (PEA^+^ = phenylethylammonium), and (R-MBA)_2_PbI_4_ (R-MBA^+^ = R-methylbenzylammonium), each exhibiting distinct structural distortions and lattice dynamics. In the case of the PA^+^ cation, as well as other alkylammonium cations, the width of the cation is smaller than the cavity formed by the four octahedra, leading to out-of-plane octahedral tilting as the temperature decreases. In contrast, the aromatic rings in the PEA^+^ cation suppress this out-of-plane tilting, and no phase transition is observed in the temperature range 80–320 K. The R-MBA^+^ cation, representing an “oversized” cation, has increased steric hindrance due to the methyl group at the α-position of the ammonium headgroup. This steric effect elongates the in-plane Pb–I bond length, causing Pb^2+^ off-center displacement along the most stretched direction. The steric hindrance of spacer cations can be quantified by the volume of the [NI_8_] polyhedron, as shown in Figure 1a. As the steric hindrance increases, the inorganic lattice expands, first reducing the out-of-plane tilting and then stretching the Pb–I bond lengths (Figure 1b), which mirrors the structural changes observed as A–cation size increases in 3D perovskites. A sharp increase in [NI_8_] polyhedron volume is noted across the phase transition of (PA)_2_PbI_4_ (Figure 1c), attributed to more dynamic disorder of the PA^+^ cation in the high-temperature phase.^45−47^

**Figure 2:**
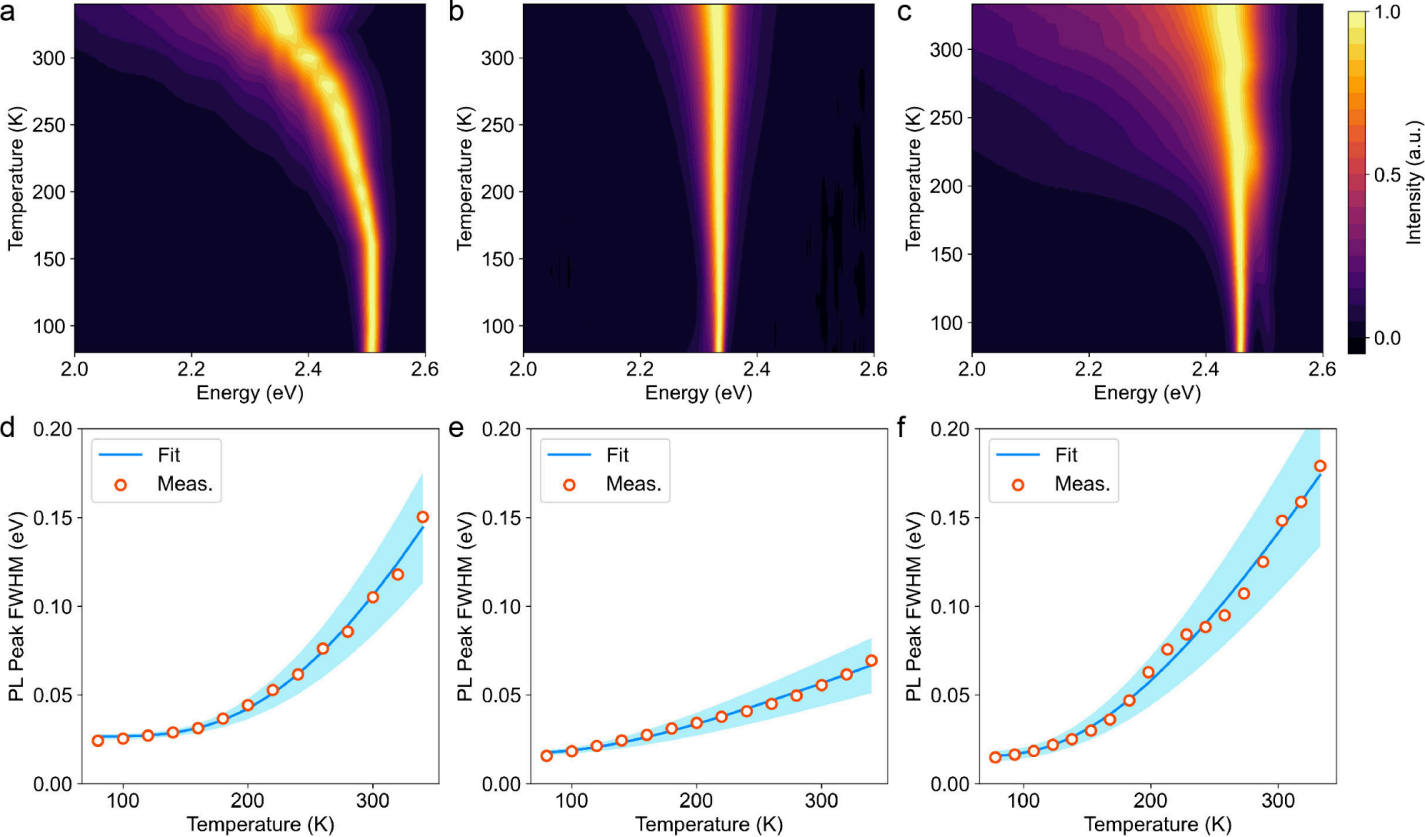
Comparison of exciton–phonon coupling in three representative 2D lead iodide perovskites. (a–c) Temperature-dependent photoluminescence spectra of (a) (PA)_2_PbI_4_, (b) (PEA)_2_PbI_4_, and (c) (R-MBA)_2_PbI_4_. (d–f) Fitting of the full-width at half-maximum (fwhm) for the excitonic peak of (d) (PA)_2_PbI_4_, (e) (PEA)_2_PbI_4_, and (f) (R-MBA)_2_PbI_4_. Blue shaded areas represent the standard deviation of the fitted fwhm at different temperatures.

Although the structural distortions in 2D perovskites are more complex than those in 3D perovskites,^48^ these three types of distortions provide a practical framework for classifying these materials and comparing their photophysical properties. Exciton–phonon coupling in semiconductors governs many important physical properties, such as carrier mobility, PL peak width, and phonon mediated nonradiative recombination.^49,50^ To quantitatively compare the exciton–phonon coupling strength, we analyze the temperature-dependent PL spectra of three representative structures (Figure 2). These structures exhibit a single PL peak from free exciton emission. At low temperatures, the PL peak width is mainly influenced by structural disorder. As the temperature increases, phonon scattering becomes the dominant factor in broadening the PL peak, which can be modeled using the following equation:^51,52^
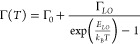
In this equation, Γ_0_ represents inhomogeneous broadening at 0 K, while the second term accounts for homogeneous broadening from longitudinal optical (LO) phonon scattering via Fröhlich interaction, with Γ_*L*0_ as the coupling strength and E_*L*0_ as the dominant or average phonon energy. Among the three structures, (PEA)_2_PbI_4_ displays the lowest Γ_*L*0_ of 0.13 ± 0.03 meV, significantly lower than the 1.8 ± 0.3 meV of (PA)_2_PbI_4_ and the 0.77 ± 0.14 meV of (R-MBA)_2_PbI_4_ (Table 1). Moreover, the PL peak energy of (PEA)_2_PbI_4_ remains almost unchanged with increasing temperature, in stark contrast to the redshift observed in (PA)_2_PbI_4_ and (R-MBA)_2_PbI_4_. These observations suggest the weakest exciton–phonon coupling in the least-distorted (PEA)_2_PbI_4_.

**Table 1:** Fitting Parameters of Exciton-Phonon Coupling in (PA)_2_PbI_4_, (PEA)_2_PbI_4_, and (R-MBA)_2_PbI_4_

LA Cation	Γ_0_ (meV)	Γ_***L***0_ (eV)	***E*_*L*_**_0_ (meV)	Fit R^2^
PA	26.5 ± 1.5	1.8 ± 0.3	82 ± 5	0.9946
PEA	17.0 ± 1.3	0.13 ± 0.03	37 ± 5	0.9924
R-MBA	15.2 ± 2.9	0.77 ± 0.14	51 ± 4	0.9920

**Figure 3:**
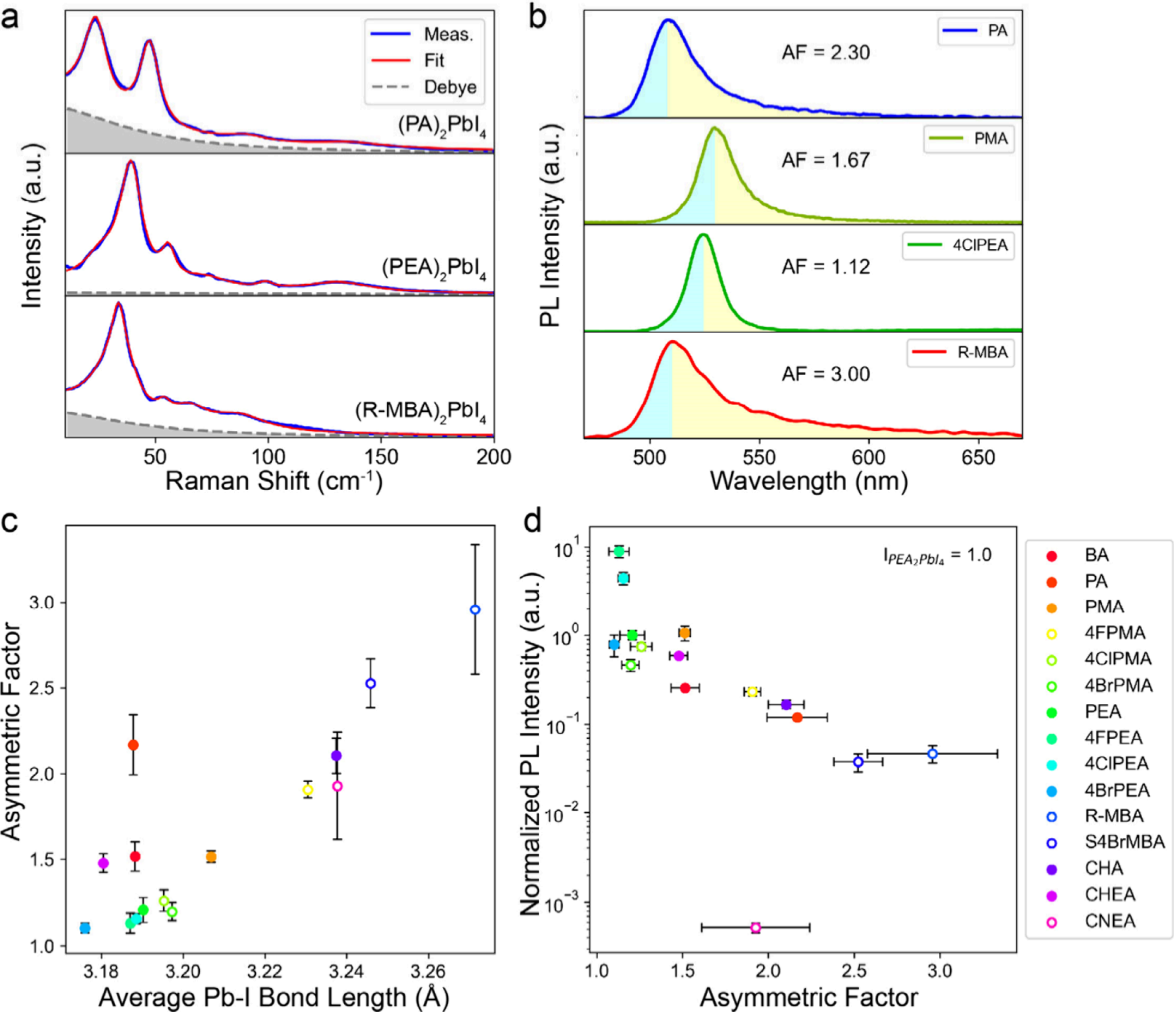
Raman and photoluminescence properties of various 2D lead iodide perovskites. (a) Low-frequency Raman spectra of (PA)_2_PbI_4_, (PEA)_2_PbI_4_, and (R-MBA)_2_PbI_4_. The gray shaded areas depict the broad central peaks from fittings. (b) PL spectra and the definition of asymmetric factor. (c) Correlations between Pb–I bond length and asymmetric factor in various 2D perovskites. (d) Correlations between asymmetric factor and relative PL intensity. The PL intensity of (PEA)_2_PbI_4_ is set to 1 as a standard. Abbreviations: BA^+^, butylammonium; PA^+^, pentylammonium; PMA^+^, phenylmethylammonium; PEA^+^, phenylethylammonium; CHA^+^, cyclohexylammonium; CHEA^+^, 2-(cyclohexyl)ethylammonium; 4XPMA^+^, 4-halophenylmethylammonium (X = F, Cl, Br); 4XPEA^+^, 4-halophenylethylammonium; CNEA^+^, 2-cyanoethylammonium; S-4BrMBA^+^, S-1-(4-bromophenyl)ethylammonium; R-MBA^+^, R-α-methylbenzylammonium. Reproduced with permission from ref (22). Copyright 2023 Wiley-VCH GmbH.

The reduced exciton–phonon coupling observed in (PEA)_2_PbI_4_, as compared to alkylammonium-based 2D perovskites, has been attributed to the greater structural rigidity of the former, as demonstrated by its substantially smaller atomic displacement parameters.^53^ It should be noted, however, that atomic displacement parameters can be influenced by several factors, including dynamic and static disorder, the quality of the crystallographic data, and the methodologies used in solving the crystal structure.^54^ Nevertheless, from a geometrical standpoint, the narrower molecular width of the alkylammonium compared to PEA^+^ allows for more available space within the interlayer gallery, leading to greater motions of the alkylammonium cation and contributing to dynamic out-of-plane octahedral tilting in the inorganic layer. The greater structural flexibility in alkylammonium-based 2D perovskites is further evidenced by a phase transition associated with out-of-plane octahedral tilting and the ordering of the alkylammonium cations as the temperature decreases.^46,47^ It is important to emphasize that the reduced exciton–phonon coupling is a consequence of the overall structural rigidity, which is governed by the packing compactness of both the organic spacer layer and the inorganic layer, rather than being related to the molecular rigidity of the spacer cation itself. For example, despite the structural similarity between PEA^+^ and R-MBA^+^, (R-MBA)_2_PbI_4_ exhibits a broader peak width than both (BA)_2_PbI_4_ and (PEA)_2_PbI_4_ at high temperatures, highlighting that the molecular rigidity of the space cation alone does not determine exciton–phonon coupling. Moreover, the comparison between (R-MBA)_2_PbI_4_ and (PEA)_2_PbI_4_ suggests exciton–phonon coupling is influenced by the lone pair distortion within the inorganic layer. The structural dynamics of the inorganic layer can further be assessed using low-frequency Raman spectra^22,55,56^ (Figure 3a). (PA)_2_PbI_4_ and (R-MBA)_2_PbI_4_ exhibit diffusive Raman peaks superimposed on a broad central peak, which are indicative of strong lattice anharmonicity and dynamic disorder.^55,56^ In contrast, (PEA)_2_PbI_4_ is characterized by several relatively well-resolved peaks and a suppressed central peak, highlighting its more rigid and ordered structure.^22,56^

To establish the structure–property relationship, the PL properties of a wider range of 2D lead iodide perovskites was investigated.^22^ The PL peaks in these materials typically exhibit an asymmetric shape with a low-energy tail, which can be quantified by the asymmetric factor (AF), defined as the ratio of integrated PL intensity on the low-energy side to the high-energy side (Figure 3b). Previous studies have established that the electron–phonon interaction is primarily responsible for the exponential tails of absorption and PL peaks in 3D perovskites.^57,58^ In 2D perovskites, it has been demonstrated that the intrinsic asymmetric PL spectrum with a low-energy exponential tail originates from momentarily trapped exciton polarons, which are characterized by an intermediate exciton–phonon interaction strength between large polaron and small polaron (or self-trapped exciton).^14,59^ A more pronounced PL tail generally indicates stronger exciton–phonon coupling and increased emission from localized excitons.^60^ It was found that both the out-of-plane octahedral tilting and lone pair distortion influence the exciton–phonon coupling. The tendency of lone pair distortion can be quantitively described by the average Pb–I bond length (*d̅*_*Pb–I*_)), with longer bond lengths indicating a reduced restoring force for B–cation displacement and thus a greater tendency for lone pair distortion.^61^ As shown in Figure 3c, AF values generally increase as *d̅*_*Pb–I*_ increases from 3.18 to 3.27 Å, correlating with the strength of lone pair distortion. However, exceptions were observed in the structures with alkylammoniums, such as (BA)_2_PbI_4_ (BA^+^ = butylammonium) and (PA)_2_PbI_4_, where large AF values occur despite relatively small *d̅*_*Pb–I*_. This stronger exciton–phonon coupling can be attributed to dynamic out-of-plane octahedral tilting. Larger AF values are generally associated with lower PL quantum yield of free exciton due to increased nonradiative recombination pathways resulting from stronger exciton–phonon coupling. This structure–property relationship was further corroborated by high-pressure studies of (R-MBA)_2_PbI_4_. With compressing its *d̅*_*Pb–I*_ from 3.27 to 3.18 Å by external pressure, the PL peak exhibits a sharp increase by a factor of 1.8 in the intenisty, along with a more symmetric shape. In Figure 3d, the PL intensity of (CNEA)_2_PbI_4_ (CNEA = 2-cyanoethylammonium) deviates from this trend, indicating that other factors, such as defect states, may also significantly influence PL intensity.

## Exciton Transport Properties

**Figure 4:**
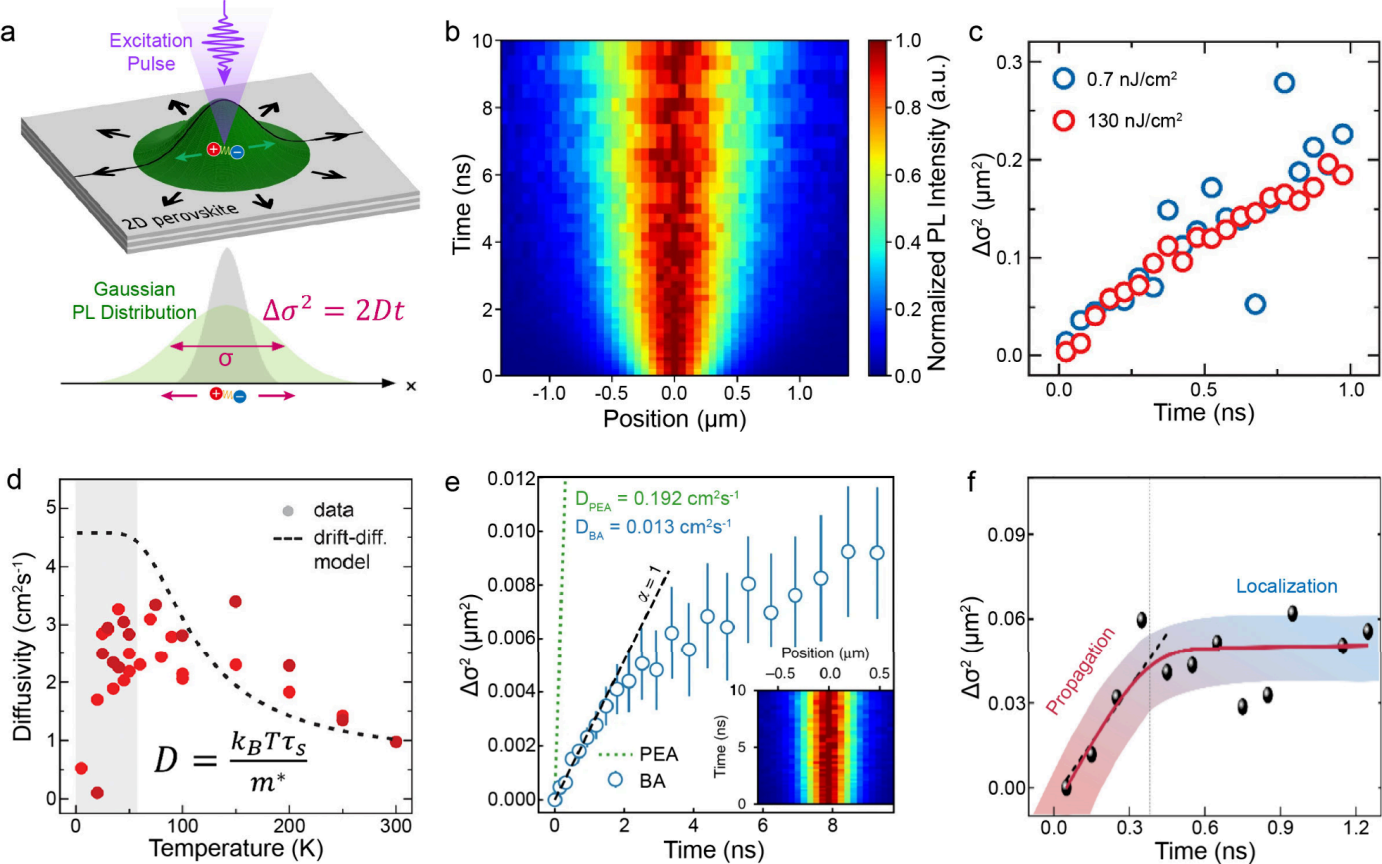
Exciton diffusion dynamics of 2D lead iodide perovskites. (a) Illustration of exciton diffusion measurement using time-resolved photoluminescence (PL) microscopy. A pulsed excitation laser is focused on the 2D perovskite flake, generating a Gaussian distribution of excitons. The subsequent diffusion and emission of excitons result in the spreading of the Gaussian profile, where the Gaussian variance σ^2^ increases linearly over time (*t*): Δσ^2^ = 2*Dt*, with *D* representing the exciton diffusivity. (b) Spatial- and time-resolved PL intensity normalized at different times after excitation, showing the spreading of the excitons. (c) Evolution of the Gaussian variance change Δσ^2^ over time in (PEA)_2_PbI_4_ flakes. (d) Temperature-dependent exciton diffusivity *D* in (PEA)_2_PbI_4_ flakes. Panels (c) and (d) are reproduced with permission from ref (24). Copyright 2020 American Chemical Society. (e) Comparison of Δσ^2^–*t* curves measured from (PEA)_2_PbI_4_ (green dashed line) and (BA)_2_PbI_4_ (blue circles). The inset shows the normalized spatial- and time-resolved PL intensity for (BA)_2_PbI_4_. Panel (a), (b), and (e) are reproduced from ref (53). Available under a CC-BY 4.0 license. Copyright 2020 Michael Seitz et al. (f) Δσ^2^–*t* curve measured from (R-MBA)_2_PbI_4_ flakes, showing a fast propagation stage followed by a localization stage. Reproduced from ref (26). Available under a CC-BY 4.0 license. Copyright 2024 Sophia Terres et al.

The above discussion indicates that the crystal structure rigidity plays a critical role in suppressing exciton–phonon coupling. As a result, 2D perovskites that minimize out-of-plane tilting and lone pair distortion, such as those based on derivatives of the PEA^+^ cation, are likely the most promising candidates for light-emitting applications, as they tend to exhibit the highest PL intensity and the most symmetric PL emission peak. Beyond light emission, carrier transport in 2D perovskites is another critical process that determines their performance in optoelectronic devices. Exciton diffusion dynamics in several 2D perovskites have been investigated mainly using spectroscopic techniques such as transient absorption microscopy (TAM) and time-resolved PL microscopy^23,24,32,33,53,62^ (TPLM, Figure 4a), which allow for the tracking the spatial distribution of PL emission or absorption signals following an excitation pulse with a diffraction-limited spot size. At the microscopic level, exciton transport is governed by factors including effective carrier mass and scattering of phonons and extrinsic defects,^63^ all of which are influenced by the choice of spacer cations.

Exciton diffusion behaviors in the two most commonly studied 2D perovskites, (PEA)_2_PbI_4_ and (BA)_2_PbI_4_, have been extensively studied. Seitz et al. demonstrated that excitons in (PEA)_2_PbI_4_ initially undergo rapid normal diffusion within the first nanosecond, followed by a slower, subdiffusive regime as excitons become trapped^53,64^ (Figure 4b,c). These trap states can be filled by applying a background illumination, which generates a steady distribution of excitons, effectively eliminating trap-limited diffusion for at least the first 10 ns. The diffusivity was measured to be 0.192 cm^2^/s at 298 K and was found to decrease with increasing temperature, indicting a band-like transport mechanism. In a separate study, Ziegler et al. reported exciton diffusivity in (PEA)_2_PbI_4_ reaching up to about 1.0 cm^2^/s at 290 K.^24^ The discrepancy between these studies may be attributed to differences in the crystal quality of the samples, which are influenced by the growth and encapsulation methods.^65−67^ According to the semiclassical band-like propagation model,^24^ the diffusivity *D* is proportional to the temperature *T*, effective exciton mass m*, and scattering rate *τ*_*s*_, expressed as . The scattering time *τ*_*s*_ can be derived from the PL peak line width using the relation . It appears that this simple model using the experimentally determined scattering rates and a computed effective exciton mass notably captures both the overall temperature dependence and the experimentally measured absolute values of the diffusivity (Figure 4d), suggesting that the rapid room temperature diffusion, on the order of 1 cm^2^/s, likely approaches the intrinsic limit of diffusivity, determined by exciton–phonon scattering. A recent work also revealed phonon-limited exciton diffusion in 2D tin iodide perovskites.^32^

In comparison to (PEA)_2_PbI_4_, the exciton diffusivity of (BA)_2_PbI_4_ is significantly lower, with measurements reported by Seitz^53^ et al. at 0.013 cm^2^/s and by Ziegler^25^ et al. at 0.46 cm^2^/s (Table 2). Several other studies have also found diffusivity values for (BA)_2_PbI_4_ in the range of 0.01 to 0.2 cm^2^/s. Given that the calculated exciton mean free path  is smaller than the exciton wave packet size, which is determined by the de Broglie wavelength , the semiclassical model is inadequate for describing the exciton transport in this material.^25,62^ As a result, additional effects, such as exciton-polarons^12^ and strong localization caused by disorder/defects,^68^ need to be considered. The coupling between excitons and lattice vibrations results in the formation of exciton-polarons, which have a heavier effective mass than the free excitons, thereby reducing diffusivity. In (BA)_2_PbI_4_, stronger exciton–phonon coupling compared to (PEA)_2_PbI_4_ may result in a heavier exciton-polaron, and thus a smaller diffusivity. Anharmonic lattice fluctuations associated with octahedral tilting, as revealed by low-frequency Raman spectra,^56^ may facilitate the formation of exciton-polaron and induce carrier localization in (BA)_2_PbI_4_ compared to (PEA)_2_PbI_4_. In addition, optical studies of (BA)_2_PbI_4_ across its phase transition revealed a nearly continuous change in the PL line width, and therefore the scattering time.^25^ However, the diffusivity remains nearly unchanged despite a 1.5-fold decrease in the effective exciton mass calculated from the average crystal structures, which suggest that the polaronic effect may be more closely associated with the dynamic structure rather than the static structure.^69^ Interestingly, it was recently demonstrated that coating (BA)_2_PbI_4_ flakes with poly(methyl methacrylate) (PMMA) significantly enhances exciton diffusivity,^62^ increasing it from 0.19 to 7.65 cm^2^/s. This improvement is attributed to the PMMA network improving lattice rigidity, which reduces exciton–phonon scattering and lattice fluctuations in the surface layers.

**Table 2:** Exciton Diffusivities of Single-Layer 2D Halide Perovskites^a^

Formula	Diffusivity (cm^2^s^–1^)	Temperature (K)	Method	Sample	Reference
(BA)_2_PbI_4_	0.058 ± 0.005	RT	TPLM	Single crystals	Xiao et al.^33^
	0.06 ± 0.03	RT	TAM	Exfoliated flakes	Deng et al.^23^
	0.013 ± 0.002	RT	TPLM	Exfoliated flakes	Seitz et al.^53^
	0.19 ± 0.04	RT	TPLM	Exfoliated flakes	Gong et al.^62^
	0.47 ± 0.28	260–272	TPLM	hBN-encapsulated exfoliated flakes	Ziegler et al.^25^
	0.46 ± 0.28	266–274	TPLM	hBN-encapsulated exfoliated flakes	Ziegler et al.^25^
	7.1–9.4	RT	TPLM	PMMA-encapsulated exfoliated flakes	Gong et al.^62^
	0.04	RT	TPLM	Polycrystalline films	Cho et al.^70^
(PEA)_2_PbI_4_	0.65 ± 0.05	RT	TPLM	Single crystals	Xiao et al.^33^
	0.227 ± 0.045	RT	TPLM	Single crystals	Magdaleno et al.^71^
	0.192 ± 0.013	RT	TPLM	Exfoliated flakes	Seitz et al.^53^
	0.204	RT	TPLM	Exfoliated flakes	Seitz et al.^72^
	1.0 ± 0.4	290	TPLM	hBN-encapsulated exfoliated flakes	Ziegler et al.^24^
	0.44 ± 0.02	RT	TPLM	PMMA-encapsulated exfoliated flakes	Gong et al.^62^
(PEA)_2_PbBr_4_	0.222	RT	TPLM	Exfoliated flakes	Seitz et al.^72^
(mFPEA)_2_PbI_4_	0.362 ± 0.002	RT	TPLM	Single crystals	Shi et al.^73^
	1.91 ± 0.09	RT	TPLM	Single crystals	Xiao et al.^33^
(R-MBA)_2_PbI_4_	0.7–2.4	290	TPLM	Exfoliated flakes	Terres et al.^26^
(2T)_2_PbI_4_	0.18	RT	TAM	Exfoliated flakes	Ou et al.^34^
(2T)_2_PbI_4_	0.32 ± 0.03	40	TPLM	Exfoliated flakes	Jin et al.^32^
	≈0	RT			
(2T)_2_SnI_4_	0.60 ± 0.08	40	TPLM	Polycrystalline films	Jin et al.^32^
	≈0.17	RT			
(3T)_2_PbI_4_	0.48 ± 0.02	RT	TAM	Exfoliated flakes	Ou et al.^34^
(4Tm)_2_PbI_4_	0.67 ± 0.03	RT	TAM	Exfoliated flakes	Ou et al.^34^
(4Tm)_2_SnI_4_	0.40 ± 0.03	40	TPLM	Polycrystalline films	Jin et al.^32^
	0.17 ± 0.03	RT			

a Abbreviations: RT = room temperature, BA^+^ = butylammonium, PEA^+^ = 2-phenylethylammonium, mFPEA^+^ = *meta*-fluorophenylethylammonium, R-MBA^+^ = R-α-methylbenzylammonium, 2T^+^ = 2-([2,2′-bithiophen]-5-yl)ethan-1-aminium, 3T^+^ = 2-([2,2’:5′,2’’-terthiophen]-5-yl)ethan-1-aminium, 4Tm^+^ = 2-(3′’’,4′-dimethyl-[2,2’:5′,2’’:5′’,2’’’-quaterthiophen]-5-yl)ethan-1-aminium.

The investigation on exciton diffusion in 2D perovskites with strong lone pair distortion remain limited, likely due to the typically weak PL emission from the free excitons in these structures.^22^ Nevertheless, a recent study by Terres et al. on (R-MBA)_2_PbI_4_ reveals that excitons in this structure exhibit two distinct diffusion regimes: an initial phase of rapid propagation within the first 300 ps, followed by subsequent localization^26^ (Figure 4f). This behavior stands in stark contrast to that observed in (PEA)_2_PbI_4_ by the same research group,^24^ which exhibits normal diffusion within the first nanosecond (Figure 4c). Moreover, at a similar low excitation fluence, where the impact of exciton–exciton annihilation is negligible,^23^ the diffusivity of (R-MBA)_2_PbI_4_ was found to be about 0.4 cm^2^/s, which is considerably lower than that of (PEA)_2_PbI_4_. Additionally, the PL lifetime of (R-MBA)_2_PbI_4_ is shorter. We hypothesize that the differences in exciton diffusion behaviors observed between these two structures may be associated with the lone pair distortion, which could lead to exciton localization. Exciton self-trapping has been observed in several 2D perovskites with significant lone pair distortion.^20,61,74,75^ Further investigation is needed to better understand the role of lone pair distortion in exciton diffusion in 2D perovskites.

**Figure 5:**
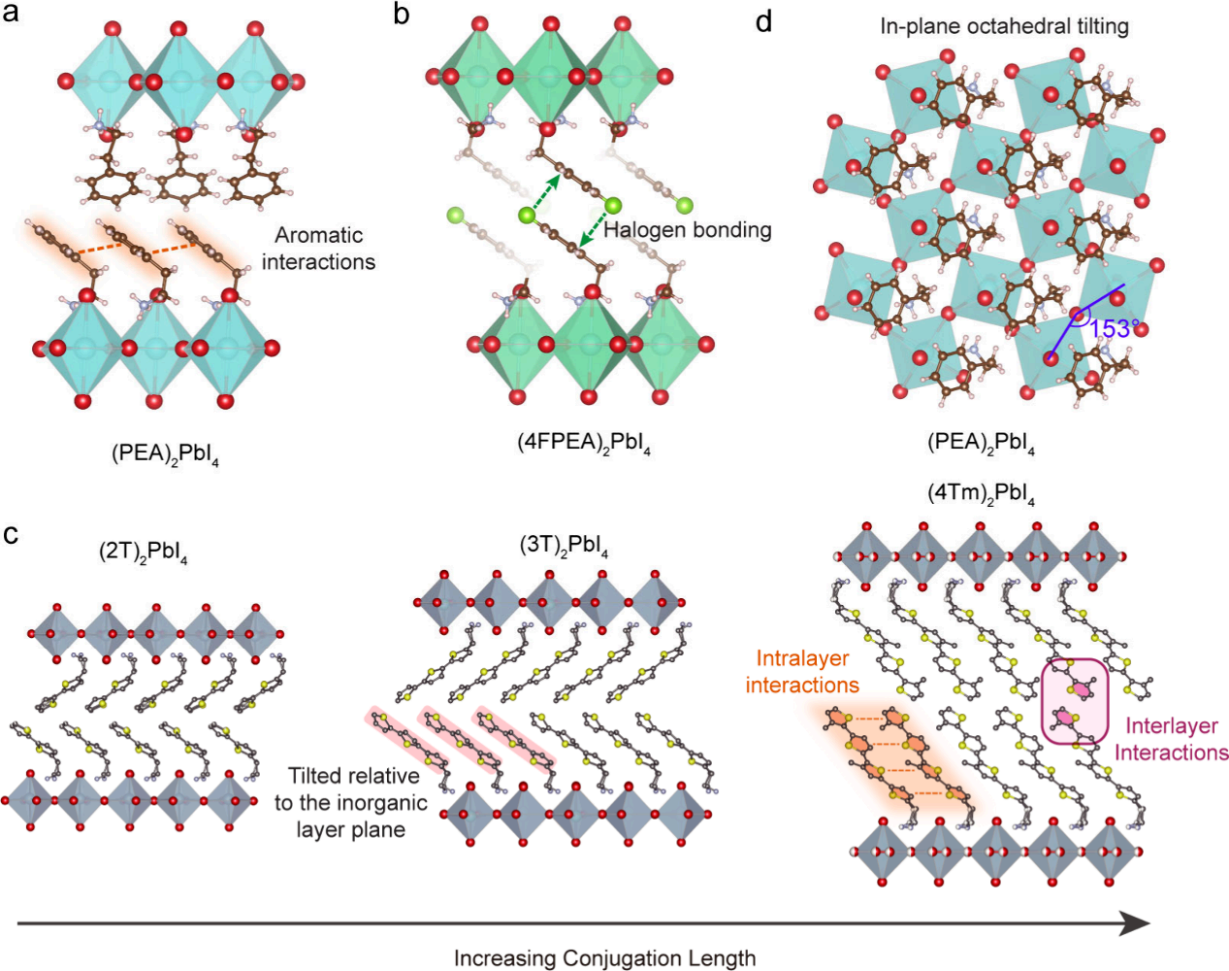
Spacer cation packing in various 2D lead iodide perovskites. (a) Crystal structure of (PEA)_2_PbI_4_ viewed along in-plane direction, highlighting the packing orientations and aromatic interactions of the phenyl rings in PEA^+^ cations. (b) Crystal structure of (4FPEA)_2_PbI_4_ viewed along the in-plane direction, with green arrows indicating the halogen bonding interactions (i.e., F···π interactions) arising from the introduction of the F atoms. (c) Crystal structures in conjugated thiophene-based 2D perovskites (2T)_2_PbI_4_, (3T)_2_PbI_4_, and (4Tm)_2_PbI_4_, highlighting the packing orientations and intermolecular forces of the spacer cations. (d) Crystal structure of viewed along the out-of-plane direction, highlighting the in-plane octahedral tilting angle of 153°. 2T^+^ = 2-([2,2′-bithiophen]-5-yl)ethan-1-aminium, 3T^+^ = 2-([2,2’:5′,2’’-terthiophen]-5-yl)ethan-1-aminium, 4Tm^+^ = 2-(3′’’,4′-dimethyl-[2,2’:5′,2’’:5′’,2’’’-quaterthiophen]-5-yl)ethan-1-aminium.

It should be noted that the photophysical properties of 2D perovskites are significantly affected by the crystal quality, particularly in terms of traps and crystalline defects. As highlighted in Table 2, large variations in exciton diffusivity have been reported by different research groups, even for the same perovskite structure. In some cases, measurements from the same group show a relative uncertainty of up to 40%, likely due to differences in crystal quality, even within the same batch of samples.^24^ It has also been demonstrated that variations in crystal growth conditions can lead to different defect densities, resulting in broadband emission in addition to free exciton emission in (PEA)_2_PbI_4_.^65,66,76^ These defects are expected to influence both the PLQY of free exciton emission and exciton diffusivity. Therefore, when comparing different structures, it is crucial to ensure that samples with minimal defects are used. Minimizing the influence of defects is essential for accurately assessing the intrinsic photophysical properties of 2D perovskites. Among the various 2D perovskites investigated, those based on PEA^+^ or its derivatives appear to be the most efficient exciton diffusion (Table 2). It is noted that while the absolute diffusivity for the same structure varies between research groups, comparative studies conducted by the same research groups consistently reveal higher diffusivity for (PEA)_2_PbI_4_ compared to (BA)_2_PbI_4_. The inclusion of aromatic rings in PEA^+^, in contrast to alkyl chains, enhances the intermolecular forces, thereby facilitating self-assembly into specific configurations that contribute to the increased structural rigidity. Although the PEA^+^ cations exhibit disorder in the crystal structure of (PEA)_2_PbI_4_, the relative orientation between adjacent cations can still be determined through refinement processes.^20^ Within each layer, the aromatic rings are aligned parallel to one another, while between layers, the rings adopt a face-to-edge (and possibly face-to-face configurations, depending on the structural model used for refinement,^29,77^Figure 5a). The plane of the aromatic ring is tilted relative to the inorganic layer to achieve denser packing. These configurations are a consequence of aromatic interactions, including π–π stacking and van der Waals forces, which are common in organic molecular solids.^78^ Substitutions on the aromatic ring that do not significantly alter the packing arrangement but enhance intermolecular interactions can further increase structural rigidity. For instance, the introduction of fluorine atoms onto the phenyl ring of PEA^+^ facilitates halogen bonding between interlayer spacer cations^79,80^ (Figure 5b), resulting in even more rigid lattices and greater exciton diffusivities in (3FPEA)_2_PbI_4_ and (4FPEA)_2_PbI_4_ (3FPEA^+^ = 3-fluorophenylethylammonium, 4FPEA^+^ = 4-fluorophenylethylammonium).^33,53^

A wide range of organic cations, including both commercially available and newly synthesized molecules, have been utilized to form 2D perovskites.^5,20^ Notably, conjugated thiophene-based spacer cations have demonstrated reduced exciton–phonon coupling and superior lasing performance compared to alkylammonium cations.^54,81,82^ These spacer cations, which incorporate multiple aromatic rings along their length, are arranged at a tilt relative to the plane of the inorganic layer (Figure 5c). This orientation enhances intermolecular interactions and packing density, thereby contributing to enhanced structural rigidity. While such structures exhibit improved photostability and material stability,^54,81^ they still exhibit slower exciton diffusivity and wider PL peak width than (PEA)_2_PbI_4_ (Table 2). Additionally, several structural descriptors have been proposed to correlate the optical properties with crystal structures.^21,22,27,83^ For example, a recent study on 2D bilayer Ruddlesden–Popper perovskites with alkylammonium cations have demonstrated that the PL asymmetric factor correlates with the packing density of the spacer cation, with denser packing enhancing PL properties.^83^

## Conclusion and Outlook

Given the preceding discussion, one might wonder whether PEA and its derivatives are the optimal cations for 2D lead iodide perovskites. From the perspective of enhancing structural rigidity, achieving close atomic packing is essential. This requires two conditions: the presence of a perfect, nondistorted inorganic layer and an organic layer with minimized voids to restrict spacer cation motion. Distortions within the inorganic layer can lead to larger bandgaps and increased effective exciton masses, which tend to cause exciton localization and reduce diffusivity. While (PEA)_2_PbI_4_ shows minimal out-of-plane octahedral tilting, in-plane octahedral tilting and associated static disorder are evident in the solved single crystal structures. This suggests that the packing arrangement of the PEA^+^ cation remains somewhat undersized relative to the 2D lead iodide framework (Figure 5d). Ideally, a self-assembled organic framework with dimensionality precisely matching that of the nondistorted inorganic framework would be preferred, alongside the organic spacer cations exhibiting limited motions. The exploration of such optimal spacer cations for 2D perovskites demands innovative molecular designs and synthesis, which can take advantage of knowledge in the fields of organic chemistry, supramolecular chemistry, and organic crystal engineering.^78,84^ Additionally, alloying different spacer cations to fine-tune structural distortions, similar to methods used in 3D perovskites,^43^ could be another effective approach.
